# Marine Bioactive Compounds with Functional Role in Immunity and Food Allergy

**DOI:** 10.3390/nu16162592

**Published:** 2024-08-06

**Authors:** Ana G. Abril, Mónica Carrera, Manuel Pazos

**Affiliations:** 1 Department of Microbiology and Parasitology, Faculty of Pharmacy, University of Santiago de Compostela, 15898 Santiago de Compostela, Spain; agonzalez@iim.csic.es; 2 Institute of Marine Research (IIM-CSIC), Spanish National Research Council (CSIC), 36208 Vigo, Spain; mcarrera@iim.csic.es

**Keywords:** marine bioactive compounds, marine lipids, peptides, polysaccharide, food allergy, immunity

## Abstract

Food allergy, referred to as the atypical physiological overreaction of the immune system after exposure to specific food components, is considered one of the major concerns in food safety. The prevalence of this emerging worldwide problem has been increasing during the last decades, especially in industrialized countries, being estimated to affect 6–8% of young children and about 2–4% of adults. Marine organisms are an important source of bioactive substances with the potential to functionally improve the immune system, reduce food allergy sensitization and development, and even have an anti-allergic action in food allergy. The present investigation aims to be a comprehensive report of marine bioactive compounds with verified actions to improve food allergy and identified mechanisms of actions rather than be an exhaustive compilation of all investigations searching beneficial effects of marine compounds in FA. Particularly, this research highlights the capacity of bioactive components extracted from marine microbial, animal, algae, and microalgae sources, such as *n*-3 long-chain polyunsaturated fatty acids (LC-PUFA), polysaccharide, oligosaccharide, chondroitin, vitamin D, peptides, pigments, and polyphenols, to regulate the immune system, epigenetic regulation, inflammation, and gut dysbiosis that are essential factors in the sensitization and effector phases of food allergy. In conclusion, the marine ecosystem is an excellent source to provide foods with the capacity to improve the hypersensitivity induced against specific food allergens and also bioactive compounds with a potential pharmacological aptitude to be applied as anti-allergenic in food allergy.

## 1. Food Allergy: Sensitization and Effector Phases, Inflammation and Gut Dysbiosis

The World Health Organization (WHO) recognizes food allergies as a significant public health concern. Food allergies occur when the immune system reacts adversely to specific molecules found in certain foods. This immune response can lead to various symptoms, ranging from mild discomforts such as skin reactions, nausea, vomiting, and diarrhea to severe and life-threatening reactions known as anaphylaxis. The European Union (EU) and many other countries have identified 14 major food allergens that must be clearly labeled when present in food products. These 14 allergens are often referred to as the “Big 14” and include cereals containing gluten, crustaceans, eggs, fish, peanuts, soybeans, milk, nuts, celery, mustard, sesame seeds, sulfur dioxide and sulfites, lupin, and mollusks. It is essential for individuals with food allergies to avoid allergenic foods and carefully read food labels to identify potential allergens in processed products. If someone suspects they have a food allergy or has experienced an allergic reaction after consuming certain foods, they should seek medical evaluation and guidance from a healthcare professional or allergist [1].

In addition to the ability of the marine ecosystem to offer substances that could potentially alleviate the immune responses triggered by certain food allergens, it is important to note that marine products, particularly seafood proteins and peptides, have the potential to induce food allergy. Most allergic patients to fish react to the calcium-binding protein β-parvalbumin [2]; meanwhile, tropomyosin is the major allergenic protein found in shellfish (crustacean and mollusk) species [3]. These proteins can be present as different isoforms with diverse allergenicity, so patients can show clinical symptoms only with certain fish species. Additionally, chemical or conformational changes induced by food processing, such as thermal treatment, Maillard reaction, and the transformation of fish muscle into surimi-type products, highly influence allergenicity by perturbing/covering the conformational epitope, exposing the hidden epitope, forming a new epitope and/or reducing the content of the allergen [4,5]. The impact of food processing on allergenicity is difficult to predict since it is affected by various factors, such as the specific allergen, the processing parameters, and the food matrix. Thus, the effect of the Maillard reaction using glycosylation has been described to be species-dependent in fish and shellfish, a reliable method to reduce the allergenicity of milk and soy but not able to reduce the allergenicity of peanuts [4]. Food processing can also be a potential source for accidental cross-contamination with traces of allergens. Thus, the development of sensitive and fast detection methods of allergens in the final food products is highly important to completely ensure food safety for the consumer [6].

### 1.1. Sensitization and Effector Phases

Allergic sensitization is the process by which an individual’s immune system becomes hypersensitive to a specific allergen, leading to an allergic response upon subsequent exposure to that allergen. The exact mechanisms of allergic sensitization are complex and can involve various factors, including genetic predisposition and environmental influences. One significant aspect of allergic sensitization is related to the immune system’s response to allergens encountered through the gastrointestinal (GI) tract. Under normal circumstances, the GI tract aims to maintain oral tolerance [7]. This process is primarily regulated by a specialized subset of immune cells known as regulatory T cells (Tregs) [8]. The alteration of oral tolerance in the GI tract can lead to the initiation of an effector allergenic T cell reaction, primarily driven by Th2 cells.

**Figure 1:**
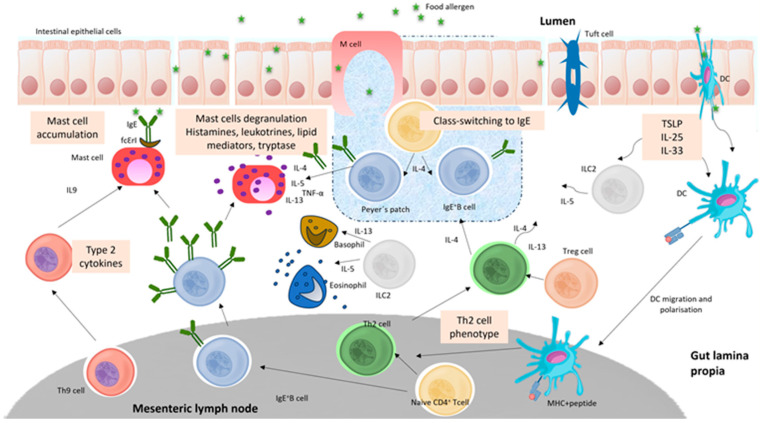
Th2 cell-mediated inflammatory response to oral antigen in the gut (adapted from Abril et al. [10]).

Effector T helper 2 (Th2) cells are a subset of CD4+ T cells that play a crucial role in regulating the immune response, particularly in allergic reactions. Th2 cells are known for their ability to produce specific cytokines such as IL4, IL5, and IL13 that are involved in the regulation of the IgE (Immunoglobulin E) response. These specific cytokines (IL4, IL5, and IL13) are essential for B cell class converting, the production of antigen-specific IgE, the extension of allergic effector cells, and the occurrence of clinical symptoms [9]. Dysregulation of Th2 responses can contribute to the development of food-allergic diseases, highlighting the importance of understanding and modulating these responses for therapeutic purposes [9]. Figure 1 summarizes the Th2-mediated inflammatory response to oral antigens. 

However, the platform has undergone high refinement in recent years, and it is too simple to include all types of immune responses. More precisely, the discovery of innate lymphoid cells (ILCs) has recently exposed a much broader landscape for the immune system, particularly in the setting of food allergies. This essay will explore the discovery of ILCs, in particular, how their functions in food allergy extend the above-described Th1 and Th2 responses [11]. In analogy to Th1, Th2, and Th17 adaptive T cell subsets, ILCs can be divided into three groups according to their cytokine profiles and transcription factor dependencies. These are ILC1, ILC2, and ILC3, correspondingly. A more specific role in food allergy pathogenesis has recently been defined for ILCs and, more specifically, ILC2s. Allergen-mediated epithelial barrier disruption triggers IL-25, IL-33, and TSLP from the latter, which, in turn, turns on the ILC2s to produce IL-5 and IL-13. The latter, IL-5, participates in eosinophil recruitment and activation, while IL-13 induces mucus production and is responsible for barrier dysfunction [12]. Furthermore, IL-13 can induce smooth muscle contraction and increased gut permeability, providing a portal of entry for additional allergens and thereby amplifying the allergic response. Targeting ILC2 or their activating cytokines, including IL-25, IL-33, or TSLP, could represent a means to intervene in allergic responses. Therapies that restore epithelial barrier function or shift the gut microbiota may also indirectly influence ILC activity and, thereby, the allergic response [13].

Several studies have also shown that epigenetics regulates key immune genes’ expression and different candidate genes, and epigenome-wide association studies have been reported in food allergy. Thus, the hypermethylation of the interferon-gamma gene (IFNG) promoter leads to the reduced expression of IFNG, promoting a Th2-skewed immune response and increasing the risk of food allergy [14]. Additionally, the hypermethylation of the IL-4 gene promoter in naive T cells and its subsequent demethylation in Th2 cells allows for precise regulation of the Th1-Th2 phenotype through epigenetic mechanisms. Thus, differentiation and synthesis of specific cytokines by the mature T cell populations is strictly controlled, with a prominent role of epigenetics [15].

### 1.2. Inflammation

The inflammatory response in food allergies involves the release of various pro-inflammatory molecules, including cytokines (such as IL-4, IL-5, and IL-13, as mentioned earlier), histamine, leukotrienes, and other mediators [9]. These molecules cause several physiological changes in the body, leading to the characteristic symptoms of food allergies. To manage food allergies effectively, individuals with food allergies must strictly avoid allergenic foods and be prepared to respond promptly with appropriate medical interventions in case of accidental exposure. Additionally, some individuals may undergo allergen immunotherapy (desensitization) under the supervision of a specialized allergist, which aims to reduce the severity of allergic reactions over time [16]. However, these treatments are still under active research and may not be suitable for everyone with food allergies.

On the other hand, non-IgE-mediated food allergies are cell-mediated immune reactions that are time-delayed [17,18,19]. The significant ones are as follows:

- Food protein-induced enterocolitis syndrome (FPIES) [20]: This is a food allergy that causes gut inflammation primarily in cow milk, soy, rice, oats, and other foods. It is marked by repetitive projectile vomiting, diarrhea, lethargy, and low blood pressure 1–4 h after the intake of food. These allergies have different immunological mechanisms and are mostly cell-mediated.
- Food protein-induced allergic proctocolitis (FPIAP) [21]: Quite common in infants, this is a condition whereby the rectum and the colon become irritated, leading to blood-streaked stools with mucus. The symptoms improve when the offending food is removed from the diet either from the infant or from the breastfeeding mother. In most cases, it involves T-cell activation and other immune pathways that are non-IgE.
- Food Protein-Induced Enteropathy (FPE) [22]: This is a chronic inflammation of the small intestine to foods, with diarrhea, vomiting, poor growth, and sometimes abdominal swelling. It usually manifests weeks after the introduction of foods like cow’s milk, soy, wheat, and eggs. The immune response is primarily cell-mediated.
- Eosinophilic esophagitis (EoE) [23]: This is a chronic esophageal disorder caused by food allergies and causes dysphagia, bolus impaction, vomiting, and abdominal pain. Diagnosis usually requires endoscopy with subsequent biopsy. The mechanism of EoE involves a chronic, immune antigen-mediated esophageal illness characterized by eosinophil infiltration. Evidence for a substantial contribution by T cells in driving an inflammatory response against food allergens is already available.

These allergies are diagnosed by history, symptom patterns, and elimination diets associated with food challenges. No specific blood or skin tests are available for these allergies. Their avoidance is based on avoiding the inciting food and sometimes medications like steroids or proton pump inhibitors in conditions like EoE. Most children outgrow these allergies by early childhood.

### 1.3. Gut Dysbiosis

Gut dysbiosis refers to an imbalance in the composition and diversity of the gut microbiota, the complex community of micro-organisms living in the gastrointestinal tract. This imbalance can result from various factors, including diet, medication use (such as antibiotics), stress, and certain medical conditions [24]. Emerging research suggests that gut dysbiosis may play a role in the development and exacerbation of food allergies [25]. When there is dysbiosis, meaning an alteration in the normal gut microbial community, it can lead to immune dysfunction and increased inflammation in the gut, potentially contributing to the development of food allergies. Several mechanisms have been proposed to explain the link between gut dysbiosis and food allergies:

Intestinal Barrier Dysfunction: A healthy gut microbiota helps maintain the integrity of the intestinal barrier, which acts as a physical and immunological barrier between the gut lumen and the body’s internal environment. Dysbiosis may compromise this barrier function, leading to increased permeability, also known as “leaky gut.” This increased permeability can allow allergenic food proteins to cross the intestinal barrier more easily, triggering immune responses and promoting the development of food allergies [26].

Immune Regulation: The gut microbiota plays a crucial role in educating and balancing the immune system. Dysbiosis can lead to an imbalance in immune responses, including an overactive Th2 response, which is associated with allergic reactions [27]. This imbalance may contribute to an increased risk of developing food allergies.

Short-Chain Fatty Acid Production: Some beneficial gut bacteria produce short-chain fatty acids (SCFAs), fatty acids of two to six carbon atoms, as byproducts of fermenting dietary fiber. SCFAs have anti-inflammatory properties through the regulation of several G-protein-coupled receptors (GPRCs) and can help regulate immune responses. Dysbiosis may reduce the production of SCFAs, leading to increased inflammation and potentially promoting food allergy development [28]. SCFAs, particularly the butyrate four-carbon SCFA, may also mediate allergy response via epigenetic mechanisms. Recent investigations showed the capacity of butyrate to inhibit allergen-induced histamine release, and this effect was associated with the inhibition of histone deacetylases (HDACs) [29]. The epigenome analysis showed that butyrate caused changes in histone acetylation in human mast cells, which subsequently led to changes in gene expression related to signaling and activation in these cells. SCFAs are also able to enhance Treg numbers and possibly functions, and this might be the central mechanism by which SCFA mediates their immunomodulatory effects [30].

Influence on Oral Tolerance: Oral tolerance is the immune system’s ability to tolerate harmless substances, such as food proteins. A balanced gut microbiota is essential for the development of oral tolerance. Dysbiosis may disrupt this process, leading to an increased likelihood of developing allergies to certain foods [31].

While there is evidence suggesting a connection between gut dysbiosis and food allergies, it is important to note that the relationship is complex and not fully understood. Researchers are actively investigating these mechanisms to better comprehend the role of gut dysbiosis in food allergies. Additionally, the use of probiotics and prebiotics to modulate the gut microbiota is an area of ongoing research as a potential approach to managing or preventing food allergies [32]. However, further studies are needed before specific recommendations can be made regarding the use of probiotics for food allergy management.

The following sections will be focused on the description of the effect of several nutrients found in marine organisms, particularly marine lipids, polysaccharides and oligosaccharides, vitamin D, peptides, and other components with bioactivity to regulate the immune system, inflammation, gut dysbiosis, and other factors with potential impact on the development of food allergy.

## 2. Marine Lipids

Marine lipids contain high proportions of omega-3 polyunsaturated fatty acids (*n*-3 PUFAs), eicosapentaenoic acid (EPA, 20:5 *n*-3), and docosahexaenoic acid (DHA, 22:6 *n*-3). EPA and DHA are not produced in significant amounts by the human body [33,34], and therefore, the direct intake of seafood and/or the consumption of nutraceutical supplements enriched in marine lipids, either from fish oil or in non-fish sources, constitutes an essential supply to incorporate these fatty acids into our organism. Among the non-fish sources, oil from microalgae and marine invertebrates, specifically copepods and Atlantic krill (*Euphausia superba*), are currently important sources to balance likely deficits of marine lipids in our diets [34].

**Figure 2:**
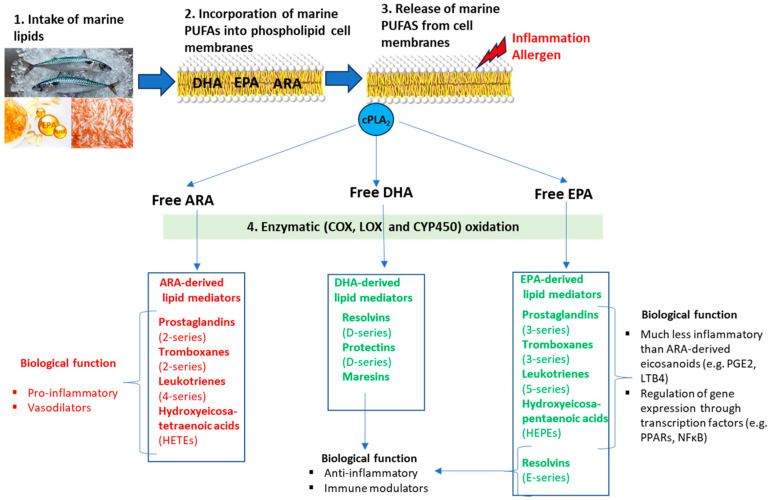
Biosynthesis of bioactive lipid mediators derived from the marine *n*-3 eicosapentaenoic acid (EPA, 20:5 *n*-3) and docosahexaenoic acid (DHA, 22:6 *n*-3) and the competition with the production of pro-inflammatory lipid mediators derived from the *n*-6 arachidonic acid (ARA; 20:4 *n*-6) (adapted from Abril et al. [10]).

From the pioneering observational studies in the Inuit Eskimo population during the 1970s and 1980s by Bang and Dyerberg [35,36], EPA and DHA have been traditionally related to a reduction in the risk of developing coronary heart disease and related cardiovascular disorders. Latter results in humans and animals have provided further evidence that dietary marine lipids can have beneficial protection to resolve different inflammatory conditions and prevent the development of several types of cancers and allergic conditions [33,37]. Many of these beneficial effects are attributed to the incorporation of marine lipids into the cellular phospholipid membranes (Figure 2). The incorporation of marine lipids into cell membranes provides a specific environment that modifies physical properties, such as fluidity, and the function of membrane proteins like receptors, signaling enzymes, and transporters. Also, importantly, EPA and DHA compete with the pro-inflammatory *n*-6 arachidonic acid (ARA; 20:4 *n*-6), either for the incorporation into membrane phospholipids or the biosynthesis for their respective *n*-3 and *n*-6 lipid-derived mediators since they share the same enzymes, as is shown in Figure 2. The consumption of a typical Western diet, which mainly consists of high-fat, high-sugar, ultra-processed foods, promotes an increase in the incorporation of ARA into phospholipid membranes. These ultra-processed foods are known for containing large quantities of ARA and its primary biosynthetic precursor, linoleic acid (LA; 18:2 *n*-6). On the contrary, the intake of marine lipids causes a replacement of the *n*-6 ARA by the *n*-3 EPA and DHA in membranes from erythrocytes and tissues [38,39].

EPA- and DHA-bound membranes are converted into essential substrates for the synthesis of the oxylipins. Oxylipins are powerful lipid mediators with bioactivity in reducing inflammation and modulating the immune system [40]. The production of oxylipins is triggered by the release of PUFAs from phospholipid membranes, primarily through the action of cytosolic phospholipase A2 (cPLA2) during normal cell regulation. However, this process is even more significant in pathological conditions like inflammation, allergy, infection, and stress. In these conditions, free EPA and DHA are metabolized to oxylipins mainly through oxidation via enzymatic processes by lipoxygenases (LOX), cyclooxygenases (COX), and cytochrome P450 (CYP450). These enzymes also metabolize *n*-6 ARA to its derived oxylipins [41]. Oxylipins derived from PUFAs with 20 carbon units in length, such as EPA and ARA, are termed eicosanoids (“*eicosa*” is Greek for 20), and the metabolites derived from the 22-carbon DHA are termed docosanoids. The principal EPA-derived eicosanoids are 3-series prostaglandins (PGs) and thromboxanes (TXs), 5-series leukotrienes (LTs), hydroxyeicosapentaenoic acids (HEPEs), and E-series resolvins (Rvs). On the other hand, the main bioactive docosanoids derived from DHA are D-series resolvins (Rvs), protectins (PDs), and maresins (MaRs) (Figure 2). The principal eicosanoids derived from ARA are 2-series prostaglandins and thromboxanes, 4-series leukotrienes, and hydroxyeicosatetraenoic acids (HETEs).

In general, it has been observed that oxylipins derived from marine lipids EPA and DHA are less inflammatory when compared to eicosanoids derived from ARA. The excessive production of ARA-derived eicosanoids, such as prostaglandin E2 (PGE2) and leukotriene B4 (LTB4), has been closely linked to the occurrence of inflammatory diseases [42]. Dietary interventions with different proportions of the marine fatty acids EPA and DHA, compared to a diet supplemented with *n*-6 LA from soybean oil, were found effective in decreasing the generation of proinflammatory ARA eicosanoids, in accordance with a reduction in inflammatory markers such as the *n*-6/*n*-3 ratio in plasma and membranes in either in a standard rat model [39] or a genetically obese hypertensive rat (SHROB) model of Metabolic Syndrome [38]. The supplementation of marine lipids has also been effective in reverting the impact of a High-Fat High-Sucrose (HFHS) diet by reducing the generation of secondary ARA metabolites, increasing the formation of secondary metabolites from EPA (11-HETE and PGE2) and reducing biomarkers of inflammation [43]. This modification of the profile of oxylipins towards the production of EPA- and DHA-derived mediators, instead of the generation of the pro-inflammatory ARA-derived eicosanoids, agreed with an improvement for parameters of inflammation, oxidative stress, and protein oxidation in animal models [39,44]. On the other hand, the intake of marine lipids has demonstrated immunomodulatory effects by changing the profile of eicosanoids produced and decreasing the levels of proinflammatory cytokines in patients with rheumatoid arthritis and inflammatory bowel disease (IBD) [42]. Although the exact causes of IBD remain unclear, several investigations suggest common etiological characteristics with allergy and also potential common treatment pathways [45].

Marine lipids are also able to modulate gut microbiota and improve gut dysbiosis by increasing the abundance and diversity of beneficial bacteria in animal models and human studies. Several investigations showed the capacity of marine lipids with high levels of *n*-3 PUFAs, particularly EPA and DHA, to modify gut microbiota and intestinal dysfunction in animals and humans [10,46]. Marine lipids affect the distribution of intestinal flora by increasing the abundance of Bifidobacteria and other beneficial bacteria while inhibiting the growth of potentially harmful bacteria like Enterobacteria [47]. Bifidobacteria and Lactobacilli, among other anaerobic bacteria, partially metabolize *n*-3 PUFAs in the distal intestine, and such a process may induce changes in the distribution of the intestinal flora, with the promotion of beneficial bacteria, such as *Bifidobacterium*, while inhibiting the proliferation of harmful bacteria [48,49]. Other results showed that fish lipid supplementation increased the abundance of gut bacteria linked to the synthesis of the beneficial SCFA butyrate [50]. These data present new evidence that suggests marine lipids possess the potential to influence the composition of the human gut microbiota, specifically targeting the presence of select advantageous bacterial taxa.

Fish oil, specifically its omega-3 PUFA component, can impact histone marks and gene expression. Studies have shown that fish oil supplementation can affect histone acetylation levels in immune genes in neonatal T cells, and maternal fish consumption during pregnancy can lead to increased acetylation in certain genes in the placenta, especially in female offspring [51].

Animal models have been utilized to explore the impact of marine lipids on the development of food allergy and inflammatory-related processes. A study conducted on mice demonstrated that feeding them with EPA, DHA, or a combination of both resulted in reduced activation and differentiation of pro-inflammatory T cells in comparison to corn oil [52]. This research also revealed a decrease in the expression of the pro-inflammatory cytokine IL-17 and the transcription factor RORγt in mice fed with fish oil without affecting Treg cell polarization. These findings suggest that marine lipids have a direct influence on the development of inflammatory T cells, potentially serving as an anti-inflammatory mechanism by suppressing this subset. Additionally, other studies have shown that fish oil can alleviate symptoms of allergies in mice sensitized to certain allergens like ovalbumin [53] and cow’s milk protein [54]. Notably, DHA-rich fish oil was found to be more effective in mitigating allergic reactions in mice allergic to certain proteins compared to EPA-rich fish oil [55]. The research indicates that consuming marine lipids can effectively reduce the outcomes of food allergy by inhibiting the activation and differentiation of T cells into pro-inflammatory types, as well as decreasing the expression of pro-inflammatory cytokines. Despite this promising evidence, there is a lack of conclusive clinical studies regarding the potential benefits of marine lipids on food allergy.

## 3. Polysaccharides and Oligosaccharides

Polysaccharides are the major components in the cell walls and extracellular matrix of seaweeds or the exoskeletons of crustaceans [56]. They present good qualities for their application in different fields, such as therapeutics, as they are biodegradable, biocompatible, nontoxic, physiologically inert, and provide the formation of hydrogels, which let them bind to other components [56]. The application of marine polysaccharides has shown enhancement of the innate immune system with a decrease in allergic responses, leading to the balance of IgE production and suppression of mast cell degranulation [57]. The main difference between marine polysaccharides and others is the presence of sulfate and amid groups [58,59]. The sulfate group is mostly found in algal polysaccharides, such as sulfated galactan [60] and sulfated fucans [61]. On the other hand, the amid group is present in chitin found in a wide range of shellfish such as shrimp, crab, oyster, squid, and marine diatoms [62]. Moreover, it was determined that the immunomodulatory effect of total extracts of polysaccharides from different marine sources directly depends on the triple-helix structure [63] and their molecular masses, such as for *Ganoderma lucidum* [64]. *Cordyceps militaris* random coil polysaccharides also demonstrated their involvement in activating macrophages and cytokine (IL-1 and TNF-α) induction [65]. The allergic activity of a sulfated polysaccharide fraction (GLSP) extracted from *Gracilaria lemaneiformis* was studied using RBL-2H3, BALB/c mice, and KU812 cells [66]. Results have shown that GLSP ameliorates allergy symptoms by decreasing TM-specific IgE, IgG1, and Th2 cell polarization and also activating KU812 while suppressing the function of RBL-2H3 cells [67]. Sulfated oligosaccharides of *Gracilaria lemaneiformis* (GLSO) were also evaluated [68]. GLSO was prepared from sulfated polysaccharides. GLSO alleviates food anaphylaxis; reduces the expression of immunoglobulin E, histamine, T helper 2 and B cells, and cytokines in ovalbumin (OVA)-sensitized mice; and up-regulates regulatory T cells (Tregs) differentiation through the maintenance of gut microbiota homeostasis [68].

Alginic acid, a linear polysaccharide consisting of 4-linked mannuronic acids and guluronic acids, is the major polysaccharide in the intracellular matrix of brown seaweeds. 

Alginate, the inorganic salt form (e.g., sodium salt) of alginic acid, plays a role as an anti-allergic compound by directly decreasing the release of histamine originating in mast cells and also by reducing hyaluronidase activity [69]. Alginates and their derivates were found to be potent immunostimulants/immunomodulators [70,71]. The presence of alginates provided the expression of histidine decarboxylase, the production of IL-1 and tumor necrosis factor-alpha (TNF-α), and stimulation of HMC-1 cells [72]. In addition, alginate oligosaccharides (AOS), which are produced by the enzymatic degradation of alginate polymers into low-molecular-weight oligosaccharides, also have the capacity to decrease serum IgE in BALB/c mice previously sensitized with beta-lactoglobulin [73,74]. Alginate extracted from marine brown algae is a polysaccharide composed of guluronate (G) and mannuronate (M) units. Several studies have shown that unsaturated guluronate oligosaccharide (GOS) prepared by enzymatic degradation can stimulate the production of nitric oxide (NO), nitric oxide synthase (iNOS), TNF-α cytokine, and Reactive Oxygen Species (ROS) in murine macrophage cells [75]. However, alginate polymers and other AOS prepared by different procedures showed lower activity. The key factors that determine the macrophage-activating ability of AOS are the molecular size, the presence of unsaturated terminal structure, and the M/G ratio. The activation of macrophages by GOS involves the nuclear factor (NF)-κB and mitogen-activated protein kinase (MAPK) signaling pathways. Proteomic analysis has confirmed that GOS-induced changes in the (NF)-κB signaling pathway as well as in pathways related to glycolysis, antioxidant activity, and inflammation, thereby improving macrophage homeostasis [76]. Other studies have suggested that GOS can improve innate immunity by promoting macrophage proliferation and cytoskeleton remodeling in macrophages [77]. GOS has also been found to up-regulate Toll-like receptor 4 (TLR4) and stimulate TLR4/Akt/mTOR, TLR4/Akt/NF-κB, and MAPK signaling pathways in macrophages [77]. These findings highlight the potential of GOS as an agent that can regulate macrophages and effectively balance the immune-inflammatory response, which is crucial for the innate immune system. 

Porphyran, a sulfated polysaccharide consisting of a linear chain of alternating 3-linked β-D-galactose and 4-linked α-L-galactose-6-sulfate residues, is found in some red seaweeds and is most frequently isolated from the genus *Porphyragenus* [78]. Several studies have shown a beneficial effect of porphyran against different allergic responses. This includes stimulating IL-1 secretion by macrophages, releasing nitrite and TNF-α, enhancing the carbon clearance activity of phagocytes [79,80,81], and increasing total leucocyte and lymphocyte counts [82]. It has been demonstrated that porphyran-derived polysaccharides and oligosaccharides can attenuate food allergy and modulate enteric microflora in mice [83]. Porphyran has shown the capacity to suppress LPS-induced activation of dendritic cells while reducing interferon-γ production [84,85]. Furthermore, Porphyra polysaccharide components PP3 and PP4 have been found to promote lymphocyte proliferation and transformation [86]. Porphyran can modulate immunity in a dose-dependent manner, and the enzymatic degradation of Porphyra polysaccharides has been shown to alter their immunomodulatory activity. Specifically, Porphyra polysaccharides degraded by peptidase were effective in enhancing the proliferation, phagocytosis, and NO secretion in a RAW264.7 macrophage cell model [87]. 

Fucoidans are a heterogenous group of polysaccharides composed mainly of the L-fucose monosaccharide and sulfate ester groups, mainly derived from brown seaweed [88,89]. Fucoidans from brown algae *Fucus vesiculosus* have demonstrated a decrease in the production of proinflammatory cytokines [90]. Moreover, *Undaria pinnatifida* fucoidans reduces IL-4 and IL-13 in bronchoalveolar lavage fluid and the antigen-specific IgE in OVA-sensitized mouse airway hypersensitivity [91]. Furthermore, other investigations showed how fucoidan may reduce IgE levels in plasma and peripheral blood mononuclear cells from OVA-sensitized mice [92] and atopic dermatitis patients [93]. Fucoidan has also demonstrated an inhibitory action on IgE production, preventing *C*-germline transcription and nuclear factor-kappa B and p52 translocation in B cells [94]. 

Chitin is a linear polysaccharide and one of the most predominant polysaccharides. The capacity of chitin for modulation of immune responses has been in vitro and in vivo evaluated. There is strong evidence that chitin acts by suppressing allergen-induced type 2 allergic responses, stimulating macrophages and other innate immune cells [95,96,97,98,99]. Moreover, chitin microparticles also induce viral-specific immunity in respiratory allergy and asthma [100] by increasing IL-12, IFN-λ, and TNF-α and reducing the production of IL-4 [101]. Chitosan resulted from the *N*-deacetylation of chitin, obtaining a polymer of *N*-acetylglucosamine [62]. This material is biodegradable, nontoxic, and displays nonallergenic properties [102]. Chitosan nanoparticles have demonstrated immune response modulation in OVA-sensitized mice [103]. Chitooligosaccharides (COS) are produced by cleaving glycosidic linkages of chitosan. COS acts as a protective agent against mast cell degranulation [104,105]. Particularly, COS (<1 kDa) showed a protective effect of OVA-induced lung inflammation in mice with asthma by reducing inflammatory parameters in the lung tissue, such as IL-4, IL-5, IL-13, and TNF-α [106].

## 4. Peptides

Bioactive peptides can be found in several sources, such as in foodstuffs, including algae, mussels, and fish, and are responsible for health benefits such as antihypertensive, antimicrobial, immunomodulatory, antioxidant, anti-allergic, and anti-inflammatory functions. This valuable potential makes them significant for pharmaceutical, food, and cosmetic industry applications and also as novel drugs and ingredients [107]. Bioactive peptides can be produced by different proteolytic enzymes to release the peptide content, and this procedure appears to be the most used for peptide production [108]. Gastrointestinal enzymes, such as pepsin and trypsin, and other enzymes from bacteria or fungus sources, such as alcalase, α-chymotrypsin, pancreatin, and thermolysin, are commonly used. Enzyme mixtures are also an option to obtain the preferred results. Micro-organisms and, more specifically, gastrointestinal microbiota can produce bioactive peptides from food proteins.

Regarding marine products, *Spirulina maxima* peptides P1 (LDAVNR) and P2 (MMLDF) resulting from enzymatic hydrolysate were previously determined by mass spectrometry analysis [109,110] and studied in vitro for their determination of anti-allergy properties (Table 1). After in vitro analysis of antigen-induction, a reduction in the histamine release was shown. In addition, an increase in levels of intracellular Ca^2+^ was displayed while suppressing mast cell degranulation. In summary, the P1 peptide was involved in the signaling pathways dependent on calcium and microtubes, and the P2 played a role in the inhibition of phospholipase Cγ and the formation of ROS [110,111]. In addition, other peptides obtained from *Spirulina maxima* (ADSDGK) showed anti-allergic activity by reducing mast cell degranulation induced by egg albumin in guinea pigs. This anti-allergic effect was similar to that of the clinically used anti-allergic drug disodium cromoglycate [112].

Additionally, abalone (*Haliotis discus hannai*) intestine gastrointestinal digests were proposed with anti-allergic activity [113]. In this sense, the peptide PFNQGTFAS with MW of 1175.5 Da decreases the production of pro-inflammatory cytokines and also the histamine release after antigen induction. This peptide has been described as a candidate for therapeutics in allergy to mollusks [113].

Moreover, Atlantic salmon by-product hydrolysate peptides were identified through LC/MS/MS [84,114]. Different fractions showed variations in anti-allergic activity. The fraction with the strongest anti-allergic activity contained the TPEVHIAVDKF peptide. This novel peptide acts to inhibit the release of β-hexosaminidase in IgE-mediated RBL-2H3 cell degranulation. Other anti-allergic peptides had been identified in enzymatic hydrolysates from Atlantic salmon (*Salmo salar*) byproducts and from other fish species [84,114,115,116].

Despite these studies, the anti-allergic properties of marine peptides should be further studied and exploited because of their potential for inflammatory responses [107]. Moreover, marine protein hydrolysates are employed for several industrial purposes, while bioactive peptides individually are not often applied. An important challenge to integrate the bioactive peptides in food matrices is that they must be stable and able to resist external factors maintaining bioactivity. Encapsulation may be a solution to overcome this limitation and increase the bioactivity and stability of peptides [107]. 

## 5. Vitamin D

Vitamin D is a fat-soluble vitamin that plays a crucial role in various physiological processes, including bone health, immune function, and inflammation regulation. There is growing interest in the potential relationship between vitamin D status and the development or management of food allergies [117]. Several mechanisms have been proposed to support the link between vitamin D and food allergies:

Immune System Modulation: Vitamin D is known to modulate the immune system, and it can influence the balance between pro-inflammatory and anti-inflammatory responses. Adequate vitamin D levels may promote a more balanced immune response and help prevent excessive inflammatory reactions, which could be beneficial in managing allergic reactions, including food allergies [118].

Regulation of T-Cells: Vitamin D has been shown to influence the differentiation and function of T-cells, a type of immune cell involved in allergic responses. It may promote the development of regulatory T-cells (Tregs), which play a key role in maintaining immune tolerance and preventing excessive immune responses against harmless substances, such as food proteins [119].

Epithelial Barrier Function: Vitamin D can help maintain the integrity of epithelial barriers, including the intestinal barrier [120]. A healthy intestinal barrier is essential for preventing the entry of allergens into the bloodstream and reducing the risk of allergic reactions to food proteins.

Antimicrobial Effects: Vitamin D has antimicrobial properties and can enhance the gut’s defense against harmful micro-organisms [121]. A balanced gut microbiota is believed to play a role in food allergy prevention, and vitamin D’s effects on gut health may indirectly influence the risk of developing food allergies.

**Figure 3:**
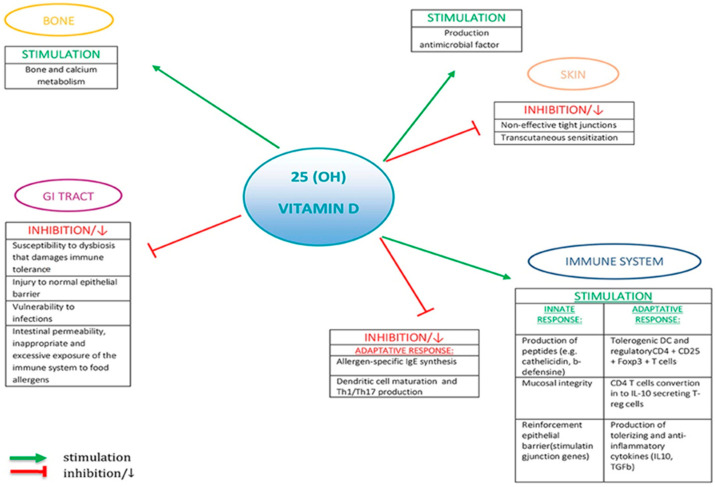
The role of vitamin D in food allergy. Reproduced from Giannetti et al., 2020 [122], with permission from publisher Frontiers, 2024 (Creative Commons CC-BY licence (CC-BY 4.0).

Despite these potential mechanisms, the relationship between vitamin D and food allergies is complex and not fully understood [122]. Research in this area is ongoing, and results from studies have been somewhat mixed. Some studies have suggested an association between lower vitamin D levels and an increased risk of food allergies. Figure 3 summarizes the role of vitamin D in food allergy.

## 6. Other Components

Other compounds from marine sources have been successfully evaluated as substances with anti-allergic effects. Brown algae provide many pigments that have immunomodulating effects in allergy. The carotenoid-derived pigments peridinin and fucoxanthin, isolated from *Symbiodinium* sp. and *Petalonia fascia*, were found to reduce the migration of ear eosinophils to eotaxin. The results showed better activity for peridinin than fucoxanthin, suggesting the potential use of peridinin as a drug to treat allergic reactions [123]. Phycocyanin is another pigment mainly found in cyanobacteria, red algae, and cryptophyte algae [115]. This pigment inhibited allergic outcomes and inflammation in mouse models of allergy and inflammatory response [124,125]. Isolated R-phycocyanin from marine *Porphyra haitanensis* decreased the production of IgE and histamine in mice with allergic reactions induced by tropomyosin, the major allergen of shellfish [126]. In addition, Spirulina content was also effective in reducing IgE levels in the serum of mice immunized with a crude shrimp extract [127]. 

Polyphenols from marine organisms have also shown the capacity to modulate food allergy, although most of the studies on the anti-allergic effects are mainly focused on polyphenols obtained from terrestrial sources, such as catechins and flavonoids [128]. Algal polyphenols, collectively referred to as phlorotannins and derived from edible brown algae, *Ecklonia* sp., and *Eisenia* sp., have proved to have anti-inflammatory and anti-allergic effects [129]. This study reported the effects of phlorotannins reducing mast cell degranulation and histamine release, attenuating the enzymatic activity of enzymes involved in the AA cascade (PLA2, COX-2, 5-LOX…), reducing signaling pathways involved in inflammatory responses (MAPK and NF-κB) and inducing changes in the immune system by regulating lymphocytes and other immunological factors, including cytokines and chemokines. 

Marine animals, such as sponges, mollusks, sea cucumbers, and corals, were also evaluated as anti-allergy compounds [130]. Four terpenoids isolated from *Dysidea villosa* were able to inhibit the β-hexosaminidase, an enzyme secreted from mast cells via antigen-induced degranulation that has frequently been used as an indicator of anaphylactic reactions, and the generation of LTB 4 and IL-4 in RBL-2H3 mast cells [131]. Furthermore, three diterpenoids isolated from the sponge *Hippospongia lachne* suppressed the production of IgE-stimulated RBL-2H3 cells [132]. Moreover, the sterol-like compound called IZP-94005, found in the sponge *Petrosia* sp., showed improvements in the allergic reaction by decreasing histamine release when evaluated against OVA-induced bronchoconstriction and smooth muscle contractions [133]. Shoji et al. [134] identified two new triterpenoids from the sponge *Penares incrustans* with effects in reducing anti-IgE-induced histamine release mast cells in rats. Moreover, other terpenoids were characterized as anti-allergic compounds from the sponge *Xestospongia bergquistia* [135].

Shell pigments from the green sea urchin *Strongylocentrotus droebachiensis* were anti-allergic by inducing a decrease in histamine-induced ileum contraction in guinea pigs [136]. These pigment extracts predominantly contained polyhydroxy-1,4-naphthoquinone, a compound that is suggested to be mainly responsible for the anti-allergic effect. Four polyhydroxylated sterol compounds extracted from the coral *Sinularia abrupta* also displayed inhibition effects on anti-IgE-induced histamine release from rat peritoneal mast cells [137].

Marine chondroitin sulfate (ChS) has also shown a potential to have anti-allergic effects. The anti-allergic effect of chondroitin sulfate (ChS) from several sources, such as sturgeon, shark, and ray tissues, was evaluated since the chemical structure of ChS is different from each different source [138,139]. Sturgeon ChS exerted better anti-inflammatory activity and immune modulatory effect than shark ChS. Moreover, anti-allergic properties of extracts from the species of sea cucumber *Stichopus japonicus* were also studied, and results showed that water fraction displays anti-allergic properties by reducing the antigen-induced degranulation of mast cells [140].

Marine micro-organisms also provide compounds with immunomodulation properties on food allergy. Harunari et al. [141] studied the anti-allergic capacity of the enzyme hyaluromycin, a member of the rubromycin family of antibiotics that is isolated from marine-derived *Streptomyces* sp. A polyketone from a deep-sea-derived fungus *Graphostroma* sp. suppressed histamine release and degranulation in RBL-2H3 cells. [142]. In the following studies, authors also characterized eight tetracyclic diterpenoids from *Botryotinia fuckeliana* with anti-allergic effects [143]. Viridicatol, a quinoline alkaloid isolated from the marine-derived *Penicillium* species, was effective in alleviating OVA-induced allergic symptoms [144]. Results indicated that viridicatol decreased the levels of specific IgE, mast cell proteases, histamine, and TNF-alpha, and also reduced the population of B cells in the spleen and increased the population of regulatory T cells in the spleen. Additionally, viridicatol improved intestinal barrier repair in mice by inhibiting the degranulation of intestinal mast cells. Furthermore, butenolides, a class of lactones with a four-carbon heterocyclic ring structure that is obtained from the marine-derived fungus *Aspergillus terreus*, provided a decrease in calcium ion carrier A23187 and antigen-induced degranulation [145]. 

## 7. Conclusions and Future Perspectives

The present investigation highlights the capacity of the marine ecosystem to provide compounds with the potential capacity to reduce the immunological outcomes associated with the hypersensitivity induced by specific food allergens. The effect of different substances found in marine organisms, including lipids, polysaccharides and oligosaccharides, vitamin D, peptides, polyphenols, terpenoids, and compounds of other different nature, to modulate the response of the immune system in food allergy has been discussed. Marine lipids, particularly EPA and DHA, have very consistent effects regulating key factors in food allergy, i.e., the immune system, inflammation, epigenetic regulation, gut microbiota, and dysbiosis. Thus, studies in animal models showed the capacity of EPA and DHA to reduce the differentiation of T cells and proliferation of pro-inflammatory cytokines and to modulate gut microbiota. EPA and DHA improve gut bacteria associated with the production of SCFA and dysbiosis associated with food allergy. The immunomodulatory effect of marine polysaccharides, which are characterized by the presence of sulfate and amide groups, is highly dependent on the triple-helix structure and their molecular mass. Several fucoidans, chitooligosaccharides, and sulfated oligosaccharides have shown anti-allergic effects and capacity to reduce inflammatory factors related to OVA-sensitized hypersensibility. Peptides, vitamin D, pigments, polyphenols, and micro-organisms from marine sources have also shown a potential to regulate key factors in food allergy, i.e., the immune system and inflammation, although there is not much information about their effect on gut dysbiosis associated with food allergy. Future research should be focused on systematic studies approaching the biological mechanism and safety issues in vivo, and interventional studies to confirm the beneficial role of these marine substances in improving and/or preventing the allergic response against the most relevant food allergens.

## Figures and Tables

**Table 1:** Previously described marine bioactive peptides with anti-allergic effects. Extracted from Abril et al., 2022 [108].

Source/Allergen	Peptide Sequence	Activity	Reference
*Spirulina maxima*	LDAVNR—MMLDF—ADSDGK	↓ Histamine ↓ intracelular Ca^2+^	[109,110]
Mollusk/Abalone intestine	PFNQGTFAS	↓ histamine, ↓ PCA ↓ inflammatory cytokines (TNF-α, IL-1β and IL-6)	[113]
Fish/Atlantic salmon byproduct	TPEVHIAVDKF	↓ β-hexosaminidase for IgE-mediated RBL-2H3 cell	[84,114]

↑ increase; ↓ decrease.
